# Slug, Twist, and E-Cadherin as Immunohistochemical Biomarkers in Meningeal Tumors

**DOI:** 10.1371/journal.pone.0046053

**Published:** 2012-09-24

**Authors:** Masaya Nagaishi, Sumihito Nobusawa, Yuko Tanaka, Hayato Ikota, Hideaki Yokoo, Yoichi Nakazato

**Affiliations:** Department of Human Pathology, Gunma University Graduate School of Medicine, Gunma, Japan; Hirosaki University Graduate School of Medicine, Japan

## Abstract

The overexpression of Twist and Slug and subsequent down-regulation of E-cadherin facilitate the acquirement of invasive growth properties in cancer cells. It is unclear which of these molecules are expressed in mesenchymal tumors in the central nervous system. Here, we investigated 10 cases each of hemangiopericytoma, solitary fibrous tumor, meningothelial, fibrous, angiomatous, and atypical meningiomas, and 5 cases of anaplastic meningioma for Slug, Twist, E-cadherin, and N-cadherin immunoexpression. Nuclear Slug expression was observed in 9/10 (90%) hemangiopericytomas and 5/10 (50%) solitary fibrous tumors, but not in any meningiomas, except for 1 case. Similarly, nuclear Twist expression was more extensive in hemangiopericytomas and solitary fibrous tumors than meningiomas. In contrast to Slug and Twist, the positive expression of E-cadherin was observed in 39/45 (87%) meningiomas, but not in any hemangiopericytomas or solitary fibrous tumors (*P*<0.0001). The fraction of tumor cells expressing E-cadherin in meningeal tumors was negatively correlated to those of Twist (*P* = 0.004) and Slug (*P*<0.0001). The overexpression of Slug and Twist with down-regulation of E-cadherin was characteristic findings in hemangiopericytomas and solitary fibrous tumors, but not in meningiomas. The immunohistochemical profiles of the two tumor groups may be useful as diagnostic markers in cases that present a differential diagnosis challenge.

## Introduction

Hemangiopericytoma (HPC) is a mesenchymal tumor that occurs at any site throughout the body [1]. The incidence of HPC accounts for 0.4% of all central nervous system (CNS) tumors [2], [3]. HPC in the CNS usually occurs in the meninges as a meningioma-like mass and clinically mimics meningiomas. HPC often recurs with high incidences of metastasis outside the CNS, whereas anaplastic meningiomas infrequently metastasize outside the CNS [4]. Solitary fibrous tumor (SFT) is another representative non-meningothelial tumor originating from the meninges, with clinical and pathological features that closely resemble those of HPC. The unifying term “hemangiopericytoma/solitary fibrous tumor” (HPC–SFT) was proposed in the 2006 WHO fascicle of soft tissue tumors [5]; therefore, HPC and SFT have recently been considered as histopathological spectra in a single entity.

**Figure 1:**
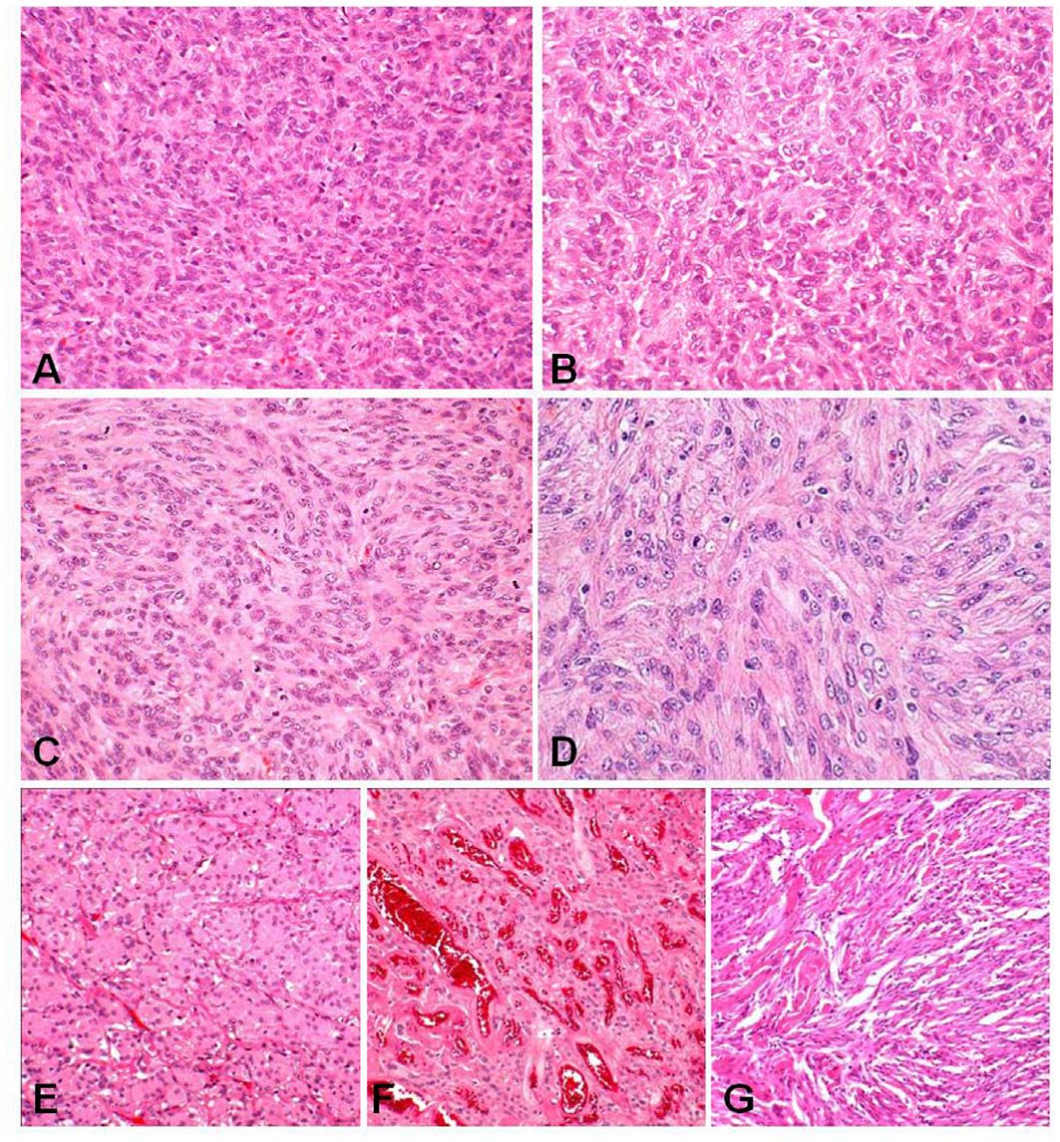
Histopathology of hemangiopericytoma, solitary fibrous tumor and meningiomas (H&E). Representative cases of selected hemangiopericytoma (A), solitary fibrous tumor (B), and, atypical (C), anaplastic (D), meningothelial (E), angiomatous (F), and fibrous (G) meningioma. Solitary fibrous tumors (A) and hemangiopericytomas (B) are occasionally difficult to distinguish from high-grade meningiomas (C, D).

HPC and SFT should be distinguished from meningiomas because of the higher rate of recurrence and later extracranial metastasis in HPC and SFT than meningiomas. However, HPC or SFT often display morphologic and immunohistochemical overlap with meningiomas, especially high-grade meningiomas (Fig. 1). Immunopositivities for vimentin and factor XIIIa are frequently observed in HPC and SFT, but are also seen in meningiomas [6], [7]. Similarly, strong diffuse immunoreactivity for epithelial membrane antigens is typical to meningiomas, but weak immunoreactivity has been reported in HPC [6], [7]. Although the expression of CD34 is a useful adjuvant to distinguish SFT and HPC from meningiomas, only 25% to 30% of meningeal HPC shows CD34 positivity [6], [7]. Utility markers clearly distinguishing these tumor types have not been found to have sufficient sensitivities and specificities.

Epithelial (E-) cadherin is an important adhesion molecule. Loss of E-cadherin leads to the breakdown of cell-cell adhesions and acquirement of invasive growth properties in cancer cells [8]. Several transcription factors including Twist and Slug induce down-regulation of E-cadherin and facilitate increased spindle morphology and cellular invasion [9], [10], [11]. It is unclear whether these transcription factors regulating E-cadherin are expressed in meningiomas, HPC, and SFT, whereas several studies have reported E-cadherin expression in meningiomas [12].

In the present study, we carried out immunohistochemistry of Slug, Twist, E-cadherin, and N-cadherin in meningiomas and non-meningothelial meningeal tumors (10 SFTs and 10 HPCs) to assess differences in expression rates of cadherins and/or transcriptional factors preceding cadherins. In meningiomas, 10 cases each of meningothelial, fibrous, angiomatous, and atypical meningiomas and 5 cases of anaplastic meningioma were examined in order to assess whether morphological differences were related to the expression of these factors.

## Materials and Methods

### Tumor samples

Formalin-fixed tissue samples, 10 each of HPC, SFT, meningothelial, fibrous, angiomatous, and atypical meningiomas, and 5 anaplastic meningiomas, were obtained from the Department of Human Pathology, Gunma University, Japan. All of these cases primarily developed on intracranial meninges and were diagnosed on the basis of the 2007 World Health Organization (WHO) Classification [3]. SFT is characterized by bland spindle cells with fibrous stroma arranged in a patternless architecture. HPC is monomorphous tumor composed of a jumbled arrangement of spindle cells with abundant reticulin fibers investing individual cells, which closely resemble histopathological findings in the cellular area of SFT. The pathology can cause confusion with high-grade meningiomas. Meningothelial meningioma is composed of uniform tumor cells forming lobules surrounded by collagenous septa. Fibrous meningioma is mainly composed of spindle-shaped cells like fibroblasts intersected by abundant reticulin and collagen fibers. Histopathological findings of fibrous meningioma overlap with those of SFT and HPC. Angiomatous meningioma is an unusual variant of meningioma, due to their abundant well-formed vessels, sinusoids, or capillaries. Atypical meningioma corresponding to WHO grade II is defined by increased mitotic activity, cellularity and small cells, patternless growth, and foci of necrosis. Anaplastic meningioma corresponding to WHO grade III exhibits obvious malignant features resembling that of carcinoma, melanoma, or high grade sarcoma (Fig.1).

### Ethics Statement

All clinical samples were procured from the Pathology Archive of Gunma University and its affiliated hospital and analyzed according to a protocol approved by the Medical Ethics Committee of Gunma University (based on the principles detailed in the Declaration of Helsinki). All patient information associated with this study was obtained in a de-identified format.

### Immunohistochemistry

Immunohistochemistry was carried out according to the streptavidin-biotin technique using a Histofine SAB-PO kit (Nichirei, Tokyo, Japan). Briefly, paraffin sections were deparaffinized in xylene and rehydrated through a graded series of ethanol dilutions. Endogenous peroxidase activity was blocked by incubation in 0.3% hydrogen peroxidase for 10 min. After pretreatments performed according to manufacturer's instructions, sections were incubated at room temperature for one hour with primary antibody and were visualized with diaminobenzidine. Antibodies were as follows: rabbit monoclonal to Slug (9585, Cell Signaling, Boston, MA; dilution, 1∶100), mouse monoclonal to Twist (ab50887, Abcam, Cambridge, UK; dilution, 1∶100), mouse monoclonal to E-cadherin (NCL-E-Cad, Novocastra, Newcastle, UK; dilution, 1∶50), and mouse monoclonal to N-cadherin (M3613, Dako, Glostrup, Denmark; dilution, 1∶50).

Staining was defined as positive for significant nuclear (Slug, Twist), cytoplasmic, or membranous (E-cadherin, N-cadherin) immunoreactivity in neoplastic cells relative to normal control meninges stained together with samples, and was scored as: –, <10% of positive cells; +, 10–49%; ++, 50–90%; and +++, >90%. The number of neoplastic cells with immunoreactivity for Slug, Twist, E-cadherin, or N-cadherin was counted in 15–20 randomly selected fields per tumor area by light microscopy at ×400 magnification. Tissues showing deviated immunoreactive areas were estimated by the average fraction of stained neoplastic cells through the section. Cases were coded and randomized (for blinding conditions) over this analysis.

### Statistical Analyses

Fisher's exact test was used to assess group differences in the analysis of qualitative features in immunohistochemical data. Pearson's correlation was used to examine associations between two variables. We declared significance at the traditional threshold of *P*<0.05.

## Results

**Figure 2:**
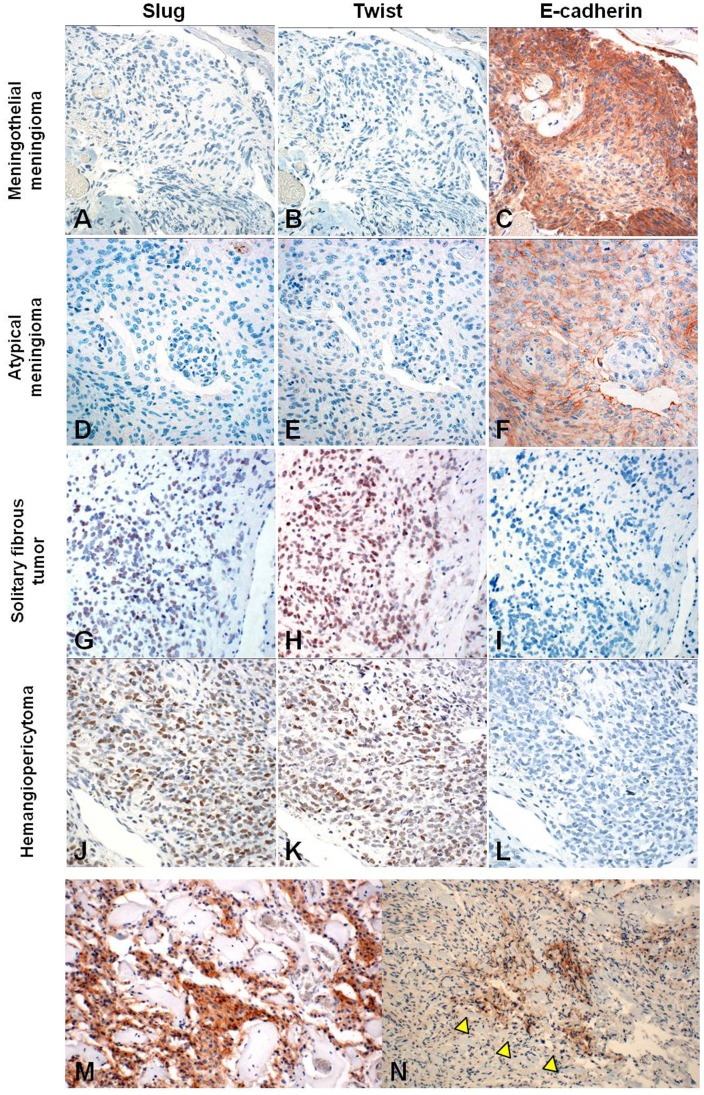
The expression of Slug, Twist, and E-cadherin in meningeal tumors. Consecutive sections of meningothelial meningioma (A–C), atypical meningioma (D–F), solitary fibrous tumor (G–I), and hemangiopericytoma (J–L). Neoplastic cells in meningothelial and atypical meningiomas are negative in immunostaining for Slug (A, D) and Twist (B, E), but are positive for E-cadherin antibody (C, F). The intensity of E-cadherin staining decreases in cases of atypical meningioma (F). In contrast, neoplastic cells in solitary fibrous tumors and hemangiopericytomas are immunopositive for Slug (G, J) and Twist (H, K), but not E-cadherin (I, L). Solitary fibrous tumors demonstrate weaker positivity to Slug (G) and stronger positivity to Twist (H) than those in hemangiopericytomas (J, K). Cytoplasmic expression of N-cadherin is more extensive in angiomatous meningiomas (M). Immunoexpression to N-cadherin is seen around bundles of collagen fibers (arrow head) in fibrous meningiomas (N).

The positive expression of Slug in ≥10% of tumor cells was restricted to SFT (5/10; 50%, *P* = 0.0004) and HPC (9/10; 90%, *P*<0.0001) cases and no meningiomas showed Slug expression, except for 1 atypical meningioma case (Fig.2, Table 1). Although Slug expression was more extensive in HPC than that in SFT cases, there was no significant difference between the two.

**Table 1:** Fraction of immunoreactive cells to Slug, Twist, E-cadherin, and N-cadherin in meningeal tumors.

	Slug	Twist	E-cadherin	N-cadherin
*Meningothelial meningioma*				
MM1	-	-	+++	-
MM2	-	-	+++	+++
MM3	-	-	+	-
MM4	-	-	-	-
MM5	-	-	++	+
MM6	-	-	+	-
MM7	-	-	+	-
MM8	-	-	+	-
MM9	-	-	++	+
MM10	-	-	++	-
*Fibrous meningioma*				
FM1	-	++	+	+
FM2	-	-	+	-
FM3	-	-	+	-
FM4	-	-	++	-
FM5	-	-	+	+
FM6	-	-	++	-
FM7	-	-	+	-
FM8	-	-	++	-
FM9	-	-	+	-
FM10	-	-	+	-
*Angiomatous meningioma*				
AngM1	-	+	++	++
AngM2	-	-	+++	+
AngM3	-	++	-	++
AngM4	-	++	-	-
AngM5	-	++	-	-
AngM6	-	-	+	+
AngM7	-	+	+++	+
AngM8	-	-	+	-
AngM9	-	++	++	+
AngM10	-	-	+	+
*Atypical meningioma*				
AtyM1	-	++	++	-
AtyM2	-	++	++	-
AtyM3	-	-	+++	-
AtyM4	-	-	+	-
AtyM5	+	-	+	-
AtyM6	-	-	++	-
AtyM7	-	-	++	-
AtyM8	-	-	++	-
AtyM9	-	-	+	+
AtyM10	-	-	++	-
*Anaplastic meningioma*				
AnaM1	-	-	+	-
AnaM2	-	-	-	-
AnaM3	-	-	+	-
AnaM4	-	-	-	-
AnaM5	-	-	+	-
*Solitary fibrous tumor*				
SFT1	+++	+	-	-
SFT2	++	+	-	-
SFT3	-	-	-	+++
SFT4	++	+	-	-
SFT5	-	+++	-	-
SFT6	-	++	-	-
SFT7	-	-	-	-
SFT8	-	++	-	+
SFT9	++	+++	-	-
SFT10	+	+++	-	-
*Hemangiopericytoma*				
HPC1	+	-	-	-
HPC2	++	+	-	-
HPC3	+	++	-	+
HPC4	+++	+++	-	-
HPC5	++	-	-	-
HPC6	++	-	-	-
HPC7	-	-	-	-
HPC8	+	+	-	-
HPC9	-	+	-	-
HPC10	++	+	-	-

The number of Slug-, Twist-, E-cadherin-, and N-cadherin-positive cells in immunohistochemistry was estimated using a four-tiered scale; -, <10%; +, 10-49%; ++, 50-90%; +++, >90%.

The positive expression of Twist in SFTs (8/10; 80%) and HPCs (6/10; 60%) in ≥10% of tumor cells was significantly higher than that in meningiomas (SFT: *P* = 0.0002, HPC: *P* = 0.017) (Fig.2, Table 1). In meningiomas, Twist expression in ≥10% of tumor cells was seen in angiomatous meningioma of 6 cases (60%), but in other meningiomas of only 3 cases (9%, *P* = 0.002).

In contrast to Slug and Twist, the expression of E-cadherin was observed in ≥10% of tumor cells in 39/45 (87%) meningiomas (9/10 meningothelial, 10/10 fibrous, 7/10 angiomatous, 10/10 atypical, and 3/5 anaplastic meningiomas), but not in any HPCs or SFTs (*P*<0.0001) (Fig. 2, Table 1). In meningiomas, expressed tumor cells in anaplastic meningiomas were seen less frequently than those in atypical meningiomas. Furthermore, there was a noticeable reduction in stain intensities for E-cadherin in atypical and anaplastic meningiomas than those in benign meningiomas (Fig. 2).

The expression of N-cadherin was largely negative in SFT and HPC. In meningiomas, over 10% of tumor cells in angiomatous meningioma expressed N-cadherin in 7/10 (70%) cases, whereas 3/10 meningothelial, 2/10 fibrous, and 1/10 atypical meningiomas revealed expression of N-cadherin in ≥10% of tumor cells (*P* = 0.001). Similar to the immunohistochemical findings of N-cadherin in angiomatous meningiomas, N-cadherin expression tended to be observed on tumor cells surrounding hyalinized vessels or bundles of collagen fibers in other tumors we investigated (Fig. 2).

Pearson's correlation coefficients indicated a positive correlation between the expression levels of Slug and Twist (*P* = 0.007) and a strong negative correlation between the expression levels of Slug and E-cadherin (*P*<0.0001) and Twist and E-cadherin (*P* = 0.004). However, there were no significant correlations between N-cadherin and Slug (*P* = 0.09), N-cadherin and E-cadherin (*P* = 0.11), or N-cadherin and Twist (*P* = 0.83).

## Discussion

E-cadherin is an important adhesion molecule that is associated with the actin cytoskeleton [13]. During a variety of biological process, E-cadherin plays a critical role in regulating the balance of cell-cell adhesion and cell motility, placing it in a significant position to regulate tumor cell proliferation and invasion [14]. Some studies have identified that reduced E-cadherin expression is associated with more aggressive epithelial tumors [15], [16], [17].

Slug is a zing-finger protein originally found to be involved in mesoderm formation, together with Twist. Slug and Twist binds the E-cadherin promoter and represses E-cadherin transcription [10], [18]. During embryonic development, neural crest cells express Slug and Twist [19], which are essential for epithelial to mesenchymal transition (EMT) [20]. In our study, meningeal tumors exhibited different expression profiles of these transcription factors and adhesion molecules. Nuclear Slug and Twist expressions were more extensive in HPC and SFT than those in meningiomas. The fraction of tumor cells expressing E-cadherin in meningeal tumors was negatively correlated to that of Twist and Slug. CD34 expression was observed in 50% HPC and 90% in SFT (data not shown). The expression of Slug and Twist was seen in most HPC cases that were negative for CD34. These results suggest that the combination of Slug/Twist/E-cadherin and CD34 staining is helpful in ultimately distinguishing HPC and SFT from meningiomas, especially from indistinguishable fibrous or high-grade meningiomas.

Overexpression of Slug and Twist has also been identified in various kinds of epithelial tumors and plays a distinct role in tumor progression via loss of E-cadherin expression [21], [22], [23], [24]. There is now evidence that these molecules facilitate tumor metastasis and tumor cell proliferation in human cancer [25], [26]. Mena et al showed that HPC metastasizes outside the CNS in 23% of cases [27], which is more frequent than in other meningeal tumors. They also reported high recurrence rates (60%) of HPC patients [27]. Dural SFT generally behaves in a benign manner and rarely has extracranial metastasis [28]. However, invasion of dural venous sinuses by intracranial SFT is relatively common [29], [30]. Through the whole SFT, 15–20% show local invasion or distant metastasis [31]. Overexpression of Slug and Twist with loss of E-cadherin may be associated with aggressive behavior in HPC and SFT.

The expression of E-cadherin was less frequent in anaplastic meningiomas and the intensity of E-cadherin staining decreased in neoplastic cells in atypical and anaplastic meningiomas without expression of Slug and Twist in our study. The down-regulation of E-cadherin has been described in high-grade meningiomas and is associated with increased tumor cell proliferation and invasive ability of meningiomas [12]. A recent study reported that loss of heterozygosity of CDH1 (E-cadherin) and nuclear localization of β-catenin was associated with down-regulation of E-cadherin in high-grade meningiomas, supporting another molecular mechanism not mediated by transcriptional factors in high-grade meningiomas that induce E-cadherin down-regulation [32].

N-cadherin is normally expressed in neuroectodermal and mesodermal-derived tissue and is involved in many processes, such as cell–cell adhesion, differentiation, embryogenesis, migration, invasion, and signal transduction [33]. De novo expression of N-cadherin associated with EMT was observed in a number of cancer types. Although a “cadherin switch” was not detected in our case, the positive expression of N-cadherin was frequently observed in angiomatous meningiomas with overexpression of Twist. It has been reported that, distinct from EMT in epithelial neoplasms, TWIST1 did not generate a "cadherin switch" [34]. Furthermore, a positive correlation was demonstrated between the expression of N-cadherin and Twist [22], [35]. These expressions may affect a weak adhesion bond among tumor cells or angiogenesis in angiomatous meningioma. On the other hand, collagen I mediates up-regulation of N-cadherin [36], which may contribute to the overexpression of N-cadherin on tumor cells surrounding hyalinized vessels and bundles of collagen fibers observed in our cases.

In summary, the overexpression of Slug and Twist with down-regulation of E-cadherin was characteristic findings in HPC and SFT. Our results suggest that these immunohistochemistries contribute to the diagnosis of meningeal tumors, especially indistinguishable meningeal tumors with atypical features.
